# Effect of repeated epilation for minor trachomatous trichiasis on lash burden, phenotype and surgical management willingness: A cohort study

**DOI:** 10.1371/journal.pntd.0008882

**Published:** 2020-12-14

**Authors:** Esmael Habtamu, Tariku Wondie, Wubshet Gobezie, Zerihun Tadesse, Bizuayehu Gashaw, Abebaw Gebeyehu, Chrissy h. Roberts, E. Kelly Callahan, David Macleod, Matthew J. Burton

**Affiliations:** 1 Clinical Research Department, International Centre for Eye Health, London School of Hygiene & Tropical Medicine, London, United Kingdom; 2 The Carter Center, Addis Ababa, Ethiopia; 3 Amhara Regional Health Bureau, Bahirdar, Ethiopia; 4 The Carter Center, Georgia, Atlanta, United States of America; 5 Department of Infectious Disease Epidemiology, London School of Hygiene & Tropical Medicine, London, United Kingdom; RTI International, UNITED REPUBLIC OF TANZANIA

## Abstract

**Background:**

WHO endorsed the use of epilation as an alternative treatment to surgery for the management of both minor unoperated TT (UTT) and postoperative TT (PTT). However, some trachoma control programmes hesitated to implement epilation citing concerns that it would hamper TT surgical acceptance and result in larger numbers of and stiffer trichiatic eyelashes than the original TT lashes. We investigated the burden and phenotypes of post-epilation trichiatic eyelashes, and willingness to accept surgical management separately in unoperated and postoperative TT cases.

**Methodology/Principal findings:**

We recruited cases with minor (≤5 eyelashes from the upper eyelid touching the eye or evidence of epilation in <1/3^rd^ of the upper eyelid) UTT (170) and PTT (169) from community-based screenings in Amhara Region, Ethiopia. Participants eyes were examined and data on present and future willingness to accept surgical management collected at baseline and every month for 6-months. Eyelashes touching the eye were counted and their phenotypes documented. Participants were trained on how to epilate. Epilation was done by the participants at home and by the examiner during follow-ups when requested by the participant. Follow-up rates were ≥97%. There was evidence of a significant reduction in the burden of trichiatic eyelashes in unoperated (mean difference = -0.90 [-1.11– -0.69]; RR = 0.50 [95% CI, 0.40–0.62]; p<0.0001), and postoperative (mean difference = -1.16 [-1.36– -0.95]; RR = 0.38 [95% CI, 0.31–0.48]; p<0.0001) cases 6-month after frequent epilation. Post-epilation trichiatic eyelashes at 6-months had higher odds of being thin (40.2% vs 55.8%, OR = 1.88 [95% CI, 1.21–2.93]; p = 0.0048), weak (39.8% vs 70.8%, OR = 3.68 [95%CI,2.30–5.88]; p<0.0001), and half-length (30.9% vs 43.3%, OR = 1.71 [1.09–2.68]; p = 0.020) than the pre-epilation trichiatic eyelashes in unoperated cases. There was a significant increase in the proportion of weak trichiatic eyelashes (OR = 1.99 [95% CI, 1.03–3.83; p = 0.039) in postoperative cases. In all 6 follow-up time points, 120/164 (73.2%) of unoperated and 134/163 (82.2%) of postoperative cases indicated that they would accept surgery if their trichiasis progressed.

**Conclusions/Significance:**

In this study setting, frequent epilation neither hampers surgical acceptance nor results in more damaging trichiatic eyelashes than the pre-epilation lashes; and can be used as an alternative to the programmatic management of minor unoperated and postoperative TT cases.

## Author summary

Trachomatous Trichiasis (TT), the blinding stage of trachoma, ranges from few peripheral eyelashes touching the eye to all eyelashes scratching the cornea. TT is mainly treated with corrective eyelid surgery. However, not all TT cases require surgical correction, and some, particularly, those with few eyelashes decline surgery. Epilation, the repeated removal of eyelashes, is a very common clinical and traditional practice in many trachoma endemic settings. The World Health Organisation recommends that epilation can be offered as an alternative management strategy to surgery for patients with few eyelashes touching the eye or refusing surgery. However, some trachoma control programmes hesitated to implement epilation with the concern that it would hamper surgical acceptance and results in larger numbers of and stiffer eyelashes touching the eye than the original TT eyelashes. In this study, we explored if these concerns are true in epilating minor (≤5 eyelashes from the upper eyelid touching the eye or evidence of epilation in <1/3^rd^ of the upper eyelid) unoperated (170) and postoperative TT (169) cases. We found, in the contrary to these concerns, the post-epilation eyelashes touching the eye were less damaging being fewer in number, thinner, weaker and shorter than the pre-epilation eyelashes. In addition, the majority of both unoperated and postoperative cases indicated that they are willing to accept surgery if their trichiasis progressed.

## Introduction

Trachomatous trichiasis (TT), the blinding stage of trachoma, has variable phenotypes ranging from a single aberrant eyelash touching the eye without entropion (the inward rotation of the eyelid margin) to complete entropion with all eyelashes in-turned towards the eye and scratching the cornea.[1] The WHO recommends surgery to reduce the risk of blindness from TT.[2] However, delivering surgical services has considerable challenges including low acceptance of surgery and poor surgical outcomes. Many individuals with TT, particularly those with mild disease decline surgery, even when this is provided free and close to home.[3–5] Moreover, TT surgery is not always successful. Individuals with just a few pre-operative in-turned eyelashes can end up with more severe postoperative TT or disfiguring eyelid contour abnormalities.[6–12]

There are very limited data on how postoperative trichiasis should be managed. However, there is consistent evidence from multiple clinical trials that most postoperative TT cases are mild (67% - 96%).[6,8,13] Secondary analysis of data from two recent clinical trials [6,8] showed that among those with PTT, ~90% had mild or no entropion, ~60% had two or fewer eyelashes touching the eye, and ~70% had aberrant (metaplastic or misdirected) eyelashes that may not warrant repeat surgery (S1 Table). These individuals, if offered, are likely to decline repeat surgery [9,14], and if they do accept surgery, the current practice is usually to repeat the same operation, which failed to fully correct the TT on the first occasion. Data presented in the 2^nd^ Global Scientific Meeting on TT indicates that repeat surgery leads to more unfavourable outcomes than the initial surgery.[15]

Epilation, the repeated plucking of eyelashes, has been practiced from antiquity for treating trichiasis, and is still a very common traditional practice in many trachoma endemic settings.[4,16–19] A significant number of patients who declined surgical treatment believe epilation is an adequate treatment for their trichiasis.[5] Data from a cross-sectional study indicate that a few non-entropic trichiatic eyelashes carry a lower risk of corneal opacity and visual impairment compared to entropic eyelashes [1], hence, epilation could be an alternative treatment for patients with a few trichiatic eyelashes. Performing tarsal rotation surgery probably is unnecessary for people with a few non-entropic eyelashes touching the eye.[9] There is evidence that successful epilation practiced by the patients is associated with less corneal opacity particularly in people with severe disease.[16,19]

We have previously conducted a large clinical trial with four years of follow-up that found minor TT cases can successfully epilate, and that epilation helps to control minor TT and limits its progression to major TT within 4 years in more than 97% of the cases.[9,14] In the 2nd Global Scientific Meeting on TT, the use of epilation as an alternative treatment to surgery for the management of both minor unoperated TT (UTT) and minor postoperative TT (PTT) was endorsed by the World Health Organization.[15] However, some trachoma programmes raised several concerns in the programmatic implementation of epilation as an alternative to surgery during and since this global scientific meeting in various national (in Ethiopia) and international trachoma programme review meetings (including the annual Carter Center trachoma program review). The commonly cited concerns were: (a) epilation could result in larger numbers of TT eyelashes; (b) post-epilation trichiatic eyelashes may be stiffer and more damaging to the cornea than the original trichiatic eyelashes; and (c) offering epilation as second line treatment to surgery would hamper surgical acceptance, as individuals may tend to choose the less invasive and less painful epilation over surgery. However, there is no evidence either to support or challenge these concerns.

In this cohort study, we aimed to investigate the burden and phenotypes of post-epilation trichiatic eyelashes, present and future willingness to accept surgery in epilating cases, corneal and visual acuity changes, and the impact of epilation on vision related quality of life (VRQoL), separately in unoperated and postoperative TT cases. These two groups of people were enrolled and analysed separately as they differ mainly based on their surgical service experiences which would affect their knowledge, attitude and practice on TT management.

## Methods

### Ethics statement

The study was approved by the Amhara Region Public Health Institute Research Ethics Review Committee (HRTT/01/789/2017), London School of Hygiene & Tropical Medicine Ethics Committee (14579), Emory University Institutional Review Board (IRB00100491); and was conducted in compliance with the Declaration of Helsinki and ICH-GCP. Participation in this study was after written informed consent documented in Amharic. If a participant was unable to read and write, the information sheet and consent form were read to them and their consent recorded by thumbprint, in the presence of a witness.

### Study design and participants

This cohort study was conducted in Amhara Region, Ethiopia. Participants were identified through community-based screening in four districts of West Gojam Zone. We recruited people with minor UTT and minor PTT, who had ≤5 eyelashes from the upper eyelid touching the eye or evidence of epilation in <1/3^rd^ of the upper eyelid in one or both eyes. The postoperative cases had a history of TT surgery at least in one eye. We excluded: (1) people under 16 years; (2) those who preferred immediate surgery; and (3) people with lower eyelid trichiasis but without upper eyelid trichiasis. Major TT cases (>5 eyelashes touching the eye or evidence of epilation of ≥1/3^rd^ of the upper eyelid) were referred for surgery. Eligible cases were invited to a local health facility and were informed about the study and treatment options. All eligible study participants were adults.

### Baseline assessments

Demographic and socioeconomic characteristics were recorded. Data on duration of the trichiasis, TT surgery knowledge, and epilation practices such as duration, frequency, epilation pain, thoughts on long term epilation practice were collected. Vision related quality of life (VRQoL) data were collected using the WHO/PBD-VF20 tool, which has previously been used in this population.[20–22]

**Fig 1:**
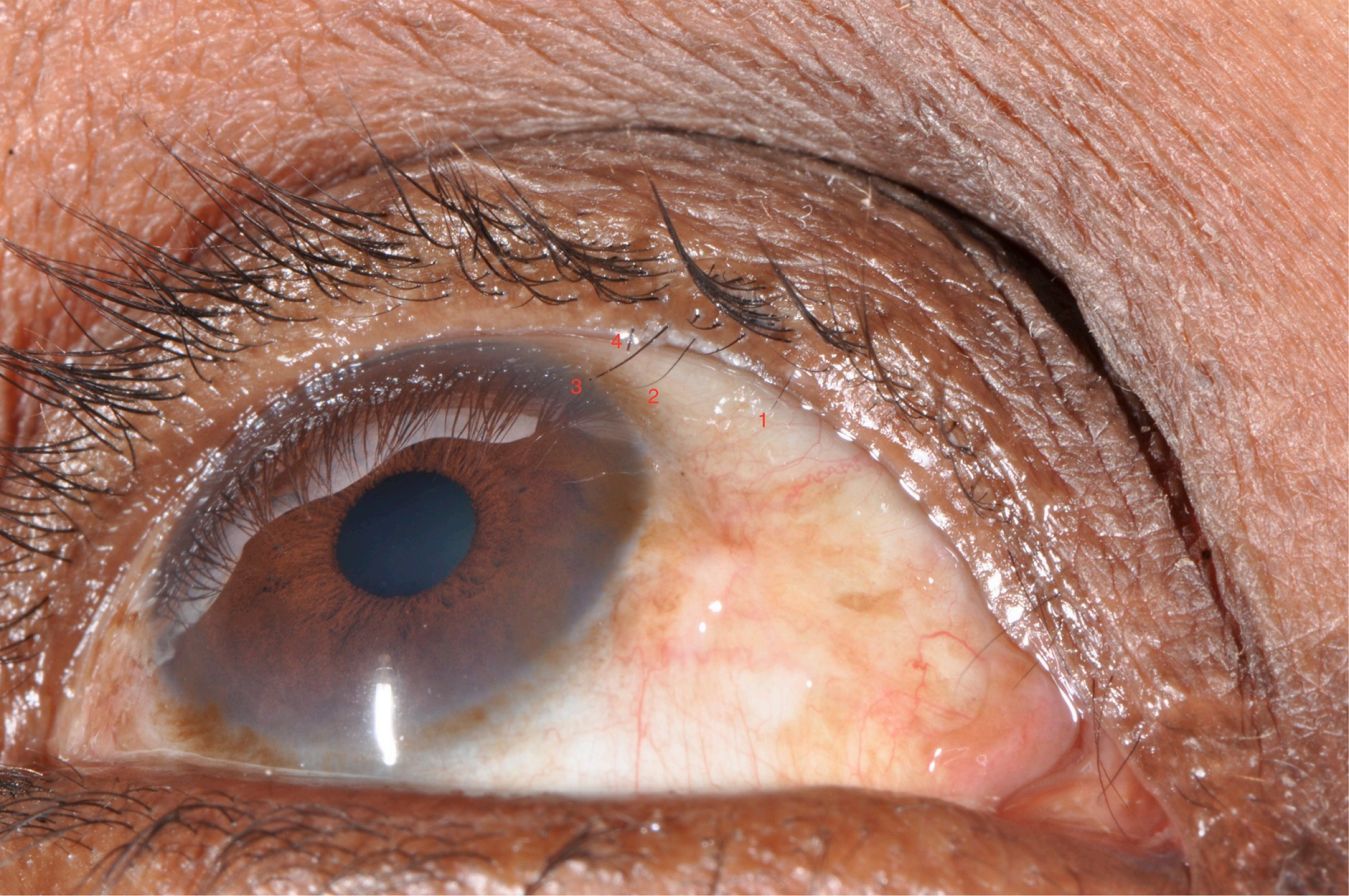
Trichiasis phenotype grading in photo.

Eyes were examined by a single examiner (WG) using 2·5× binocular loupes and torch, and graded mainly using the Detailed WHO Follicle, Papillae Cicatricea (FPC) Grading System.[23] Eyelashes touching the eye were counted and their phenotypes documented (location, thickness, strength, length, and epilation success) using a new grading system developed by our study team through rigorous piloting process, Table 1 and Fig 1. Evidence of epilation was identified by broken or newly growing eyelashes, or sections of the eyelid without lashes. Upper lid entropion was graded by assessing the degree of eyelid margin inward rotation, and grouped as mild, moderate and severe modifying a previously used grading system.[1] Corneal opacity (CO) was graded using a detailed system, based on an expansion of the FPC grading.[16] Four standardised high-resolution digital photographs of trichiasis (in primary gaze and up gaze), cornea and tarsal conjunctiva were taken (Nikon D90, 105mm macro lens).[24] Just before ocular examination, presenting logMAR visual acuity at 2 meters was measured using PeekAcuity in a dark room.[25]

**Table 1:** Trichiatic eyelash characteristics and epilation success grading.

Characteristics	Classification	Description
**Eyelash Location**^**†**^	Medial	Touching the medial conjunctiva between the medial limbus and the punctum
	Corneal	Touching any part of the cornea between the medial and the lateral limbus
	Lateral	Touching the lateral conjunctiva between the lateral limbus and the lateral canthus
Eyelash Thickness*	Thin	Trichiatic eyelashes thinner than the usual normal thickness eyelashes of the examinee
	Normal thickness	Trichiatic eyelashes of similar thickness to other non-trichiatic eyelashes of the examinee
	Thick	Trichiatic eyelashes thicker than the other non-trichiatic eyelashes of the examinee
**Eyelash Strength**	Weak	Eyelashes which touch the eyeball weakly or softly and that frequently change position with slight movements of the eyeball or are moved by tears
	Normal strength	Eyelashes that encountered the eye naturally without extra pressure or force, and that do neither move easily or encounter the eyeball firmly
	Strong	Eyelashes that directly, firmly and usually vertically encounter the eyeball, including those with sharp and stiff ends from breakage or incomplete epilation
**Eyelash Length**	Short	Trichiatic eyelashes measuring less than half (<50%) of the normal eyelash length (<5mm)
	Half (average) length	Trichiatic eyelashes measuring between ½ and 3/4^th^ (50 and 75 percent) of the normal eyelash length (about 5–7.5mm)
	Full Length	Trichiatic eyelashes approximately equal in length to non-trichiatic eyelashes, at least measure >3/4^th^ (>75% percent) of the normal eyelash length (>7.5mm)
**Epilation Success**	Complete Epilation	No visible remaining eyelash parts after epilation (eyelash removed from lash follicles/roots)
	Incomplete Epilation	Only the base of the eyelash (≤1/3^rd^) remained
	Broken	Visibly cut eyelash with >1/3^rd^ remaining

Note: Trichiatic eyelash thickness, strength and length grading was done relative to the normal (non-trichiatic) eyelashes of the examinee.

^**†**^ Trichiatic eyelash location was graded based on where it touches the eyeball in a primary position of gaze (regardless of where it originates from).

*The half of the eyelashes closest to the eye was used to grade trichiatic eyelash thickness.

After the examination, participants were trained how to epilate and advised to do this whenever they felt an eyelash touching the eye using their own traditional forceps at home and make note of the frequency of epilation and number of eyelashes epilated. Those who wanted to be epilated were assisted with epilation by the examiner (WG) using machine made epilation forceps. The amount of self-reported discomfort experienced by participant during epilation was recorded.

### Follow-up assessments

Participants were followed, interviewed, examined, and photographed monthly for 6-months following the same procedure as for baseline and by the same study team members. Data on frequency of self-epilation, number of eyelashes epilated, pain during epilation, and difficulty experienced by the participant in getting a person to epilate them were collected through interviews from the participants at each follow-up time points. Data on present and future willingness to accept surgical management were collected by asking the participants at each follow-up visit: 1) if they ‘would like to be treated with trichiasis surgery’ during the day of the follow-up, and those who wanted were referred for surgery; and 2) their ‘willingness to accept surgical management in the future if their trichiasis progressed’. Participants who received surgical management during the 6-month follow-up period were excluded from the primary analysis and other subsequent follow-up analysis. Visual acuity and VRQoL were re-measured at 1- and 6-month follow-ups.

In this study, the term “trichiatic eyelash” refers to a lash touching the eye at any follow-up time point regardless of length, thickness or strength. A post-epilation eyelash pointing to the eye but not touching it was not considered as a “trichiatic eyelash”. Participants were assisted with epilation by the examiner only if they had a lash touching the eye and success of the examiner’s epilation was documented. Masked side by side grading of the baseline and 6-months corneal photographs was done on a computer screen at about 10X magnification to determine CO progression.

### Outcomes

The primary outcome measures in both the unoperated and postoperative TT groups were (a) trichiatic eyelash burden change measured by the difference in number of trichiatic eyelashes between baseline and 6-month follow-up; (b) trichiatic eyelash phenotype change (location, thickness, strength, length) measured by the odds of change at 6-month as compared to the baseline; and (c) TT surgery acceptance during 6-month measured by the proportion of participants expressed willingness to undergo surgery. Secondary outcome measures include trichiatic eyelash burden and phenotype changes at the other follow-up time points, vision and corneal opacity (photographic grading) changes at 6-month follow-up, and VRQoL changes at 1- and 6-month follow-ups.

### Statistical analysis

A sample of 154 people was estimated to have 90% power and 95% confidence level to detect at least a 20% reduction on the burden of post-epilation eyelashes 6-month after repeated epilation. We aimed to recruit 170 unoperated and 170 postoperative TT cases to allow for about 10% loss to follow-up.

Data were double entered into Access (Microsoft), cleaned and transferred to Stata 14 (StataCorp) for analyses. Analysis was done separately on unoperated and postoperative TT cases, unless specified. For participants who had bilateral minor UTT or PTT, one eye was randomly designated to be the study eye for the analysis. Demographic and clinical history of unoperated and postoperative cases were compared using Chi square tests for categorical variables, and Wilcoxon rank sum test for continuous variables. McNemar-Bowker test of symmetry was used to test if there is a difference in the proportion of participants with the various phenotype of trichiatic eyelashes (categorical variables) between baseline and 6-month follow up separately in unoperated and postoperative cases.

The change in trichiatic eyelash burden (count data) for the primary outcome was analysed using Poisson regression to estimate rate ratio and 95% CI. The change in the mean number of trichiatic eyelashes between baseline and 6-month follow-up was analysed using random effect linear regression model after excluding prior-epilators as previous epilation may influence outcomes. Prior-epilators were defined as participants with evidence of epilation at baseline or those who reported epilating frequently at least every month in the last 6-months prior to baseline but without evidence of epilation, or those who reported epilation within the last one month prior to baseline regardless of epilation evidence. Paired t-test was used to compare mean number of trichiatic eyelashes between prior-epilators and non-prior-epilators.

The odds of post-epilation trichiatic eyelashes phenotype change and its 95% CI at the different follow-up time points versus baseline were compared using generalised linear binomial models excluding prior-epilators and only within participants who had eyelashes touching the eye at baseline that can be used as a comparison, separately for unoperated and postoperative TT cases. We compared unoperated and postoperative cases trichiatic eyelashes burden and phenotypes in all participants using Poisson regression adjusted for epilation status prior to baseline. Corneal opacity (CO) and visual acuity changes between baseline and follow-up were analysed using ordinal logistic regression. For the VRQoL, standard data analysis methods published elsewhere were employed.[21,22] The lowest possible and highest possible VRQoL scores were scaled value of 0 and 100 respectively. Then mean change in VRQoL scores between baseline, and 1- and 6-month follow-ups were analysed using a random effect linear regression model adjusted for age, sex, socio-economic status, and baseline best presenting logMar visual acuity as these factors are known to influence QoL.

## Results

### Participants enrolment and follow-up

**Fig 2:**
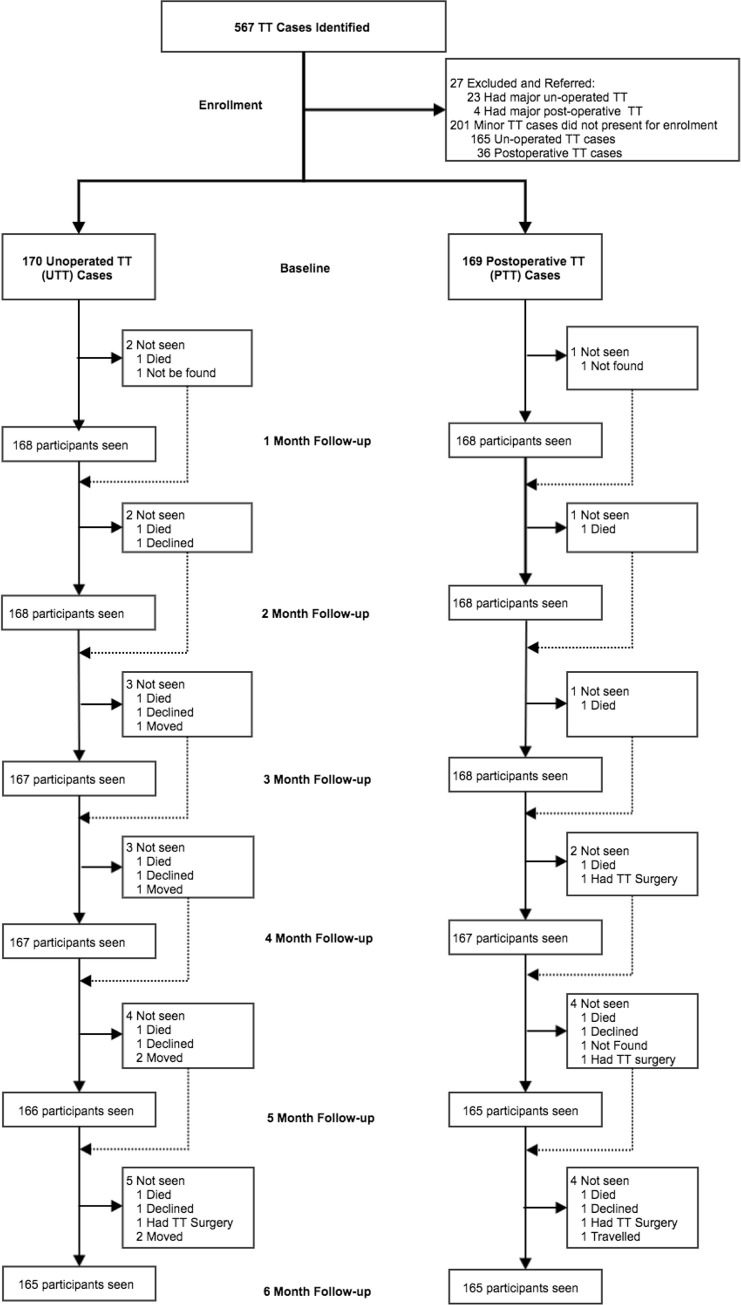
Participant flow at baseline and follow-up.

Between February 7 2018 and March 5 2018, 358 unoperated and 209 postoperative cases were identified. We excluded 23 unoperated and 4 postoperative cases with major TT. The remaining minor cases were briefed about the study and were appointed to the nearest health facility for surgery or enrolment, of whom 165 unoperated and 36 postoperative TT cases did not present. Thus 170 minor unoperated and 169 minor postoperative TT cases were enrolled. A follow-up rate of ≥97% was achieved at all follow-up time points. One participant in the unoperated TT group died prior to the 1-month follow-up, all others (338/339) attended at least one follow-up assessment, Fig 2.

### Baseline demographic and clinical history

The baseline demographic characteristics and clinical history, by TT group, are presented in Table 2. The PTT group had more females, were older, lower literacy levels and less likely to be married. A higher proportion of unoperated TT cases were unaware of their trichiasis. Comparable proportions planned to epilate; the majority responded that they would keep epilating as much as they needed. There were some differences of views on epilation, but the majority in both groups still consider epilation as an adequate treatment for their trichiasis. There were more epilators in the PTT group. Most of the unoperated TT cases were aware of surgical management for TT, and the majority of participants said they would accept surgery if their trichiasis progressed.

**Table 2:** Baseline demographic characteristics and clinical history.

Characteristic	Unoperated TT Cases N = 170		Postoperative TT Cases N = 169		P value*
	n	(%)	n	(%)	
***Gender (female)***	109	(64·1)	128	(75·7)	0.020
***Age (mean*, *SD)***	47·3	(14·2)	55.9	(12·8)	<0.0001 ^c^
***Illiterate***	140	(82·3)	156	(92·3)	0.006
***Marital status***					
Married	120	(70·6)	93	(55·0)	0.030
Widowed	24	(14·1)	34	(20·1)	
Divorced	13	(7·6)	20	(11·8)	
Single	13	(7·6)	22	(13·0)	
***Self-rated wealth***					
Very poor	13	(7·6)	16	(9·4)	0.40
Poor	63	(37·1)	66	(39·1)	
Average	84	(49·4)	83	(49·1)	
Wealthy	10	(5·9)	4	(2·4)	
Very wealthy	0	(0.0)	0	(0.0)	
***Trichiasis duration yr*, *mean (SD)***^***a***^	3.9	(4.1)	2.9	(3.2)	0.06^c^
***Prior Epilators*** ^***d***^	31	(18·2)	52	(30·8)	0.007
***Not aware presence of trichiasis***	75	(44.1)	53	(31.4)	0.015
***Epilation good enough management*?**^*‡*^					
No	24	(14·1)	42	(24.8)	0.001
Yes, definitely	85	(50·0)	90	(53.2)	
Yes, hoping	28	(16.5)	27	(16.0)	
Do not know	33	(19.4)	10	(5.9)	
***Do you think you can have epilation for long time*?**^*‡*^					
*Had no plan to epilate*	51	(30.0)	55	(32.5)	0.44
Only short term	17	(10·0)	11	(6·5)	
Long term	22	(12·9)	16	(9·5)	
Keep doing it as long as needed	80	(47.1)	87	(51.5)	
***How difficult finding epilators*** ^***b***^^*‡*^					
Easy	102	(60·4)	96	(56·8)	0.51
Difficult	67	(39·6)	73	(43·2)	
***Prior knowledge of TT surgery***^***e***^					
No	43	(25·3)	-	-	
Yes	127	(74·7)	-	-	
***Willingness to accept surgery if TT progressed*** ^***e***^					
No	41	(24·1)	42	(24.9)	0.88
Yes	129	(75·9)	127	(75.1)	

*P-values are from chi square tests

‡ Questions asked regardless of history or current epilation practice

a Trichiasis duration is analysed among those aware of the trichiasis

b Has one missing value

c P-value from Wilcoxon rank-sum test

d Prior-epilators are those who had a history or sign of epilation prior to enrolment defined as: participants with evidence of epilation at baseline or those who reported epilating frequently at least every month in the last 6-months prior to baseline but without evidence of epilation, or those who reported epilation within the last one month prior to baseline regardless of epilation evidence.

^e^ Prior knowledge of TT surgery refers to the person’s knowledge prior to being explained about surgical management for TT, while on the other hand “willingness to accept surgery if TT progressed” refers to the person’s willingness to have surgery when required in the future after being made aware that the primary management strategy to treat TT is corrective eyelid surgery.

### Trichiatic Lash Burden and Phenotype Changes

#### Unoperated TT Cases

Six-months after frequent epilation the majority (167/169) of unoperated TT cases remained minor (≤5 eyelashes touching the eye or evidence of epilation of <1/3^rd^ of the upper eyelid), 2 persons (1.22%) had >5 eyelashes touching the eye, Table 3. There were 13/169 (7.7%) cases without signs of trichiasis (had neither eyelash touching the eye nor epilation) 6-months after the baseline epilation. There was no a difference in trichiatic eyelash phenotype at baseline between those with and without sign of trichiasis at 6-months except that those without sign of trichiasis tended to have lower trichiatic lash burden at baseline than their counterparts (mean lash burden [SD] 1.46 [0.8] vs 1.87 [1.1], Wilcoxon rank-sum test p = 0.19). The mean number of trichiatic eyelashes and phenotype changes between baseline and the different follow-up time points are shown in Table 4. There was strong evidence of a reduction in trichiatic eyelash burden in unoperated TT cases between baseline and 6-month: mean lash difference = -0.90 (-1.11– -0.69); Poisson regression, RR = 0.50 (95% CI, 0.40–0.62); p<0.0001. There was strong evidence that post-epilation trichiatic eyelashes at 6-month had higher odds of being thin (p = 0.0048), weak (p<0.0001), and half-length (p = 0.020) than the pre-epilation trichiatic eyelashes at baseline, Table 4. There was no evidence of a change in trichiatic eyelashes location between baseline and the 6-month follow-up. There was no evidence of a difference in trichiatic eyelash phenotype between prior-epilators and non-prior-epilators at baseline (data not provided) except that the prior-epilators (n = 31) had significantly more average length trichiatic eyelashes than the non-prior-epilators (mean, 0.90 vs 0.55; Wilcoxon rank-sum test p = 0.035).

**Table 3:** Clinical characteristics of unoperated and postoperative TT cases at baseline and 6-month (person level).

Characteristic	Unoperated TT Cases					Postoperative TT Cases				
	Baseline N = 170		6-month N = 165		P value	Baseline N = 169		6-month N = 165		P value
	n	(%)	n	(%)		n	(%)			
***Trichiatic eyelashes burden (#)***										
0 (epilating <1/3^rd^)	0	-	73	(44.2)	<0.0001 ^a^	10	(5·9)	97	(58.9)	<0.0001^a^
1	88	(51·8)	51	(30.9)		92	(54·4)	40	(24.2)	
2	44	(25·9)	21	(12.7)		36	(21·3)	21	(12.7)	
3	21	(12·3)	11	(6.7)		11	(6·5)	5	(3.0)	
4	11	(6·5)	6	(3.6)		11	(6·5)	0	(0.0)	
5	6	(3.5)	1	(0.6)		9	(5.3)	1	(0.6)	
6+	0	-	2	(1.2)		0	-	1	(0.6)	
***Trichiatic eyelash location***										
Corneal only	120	(70·6)	65	(39.4)	<0.0001 ^b^	100	(59·2)	44	(26.7)	<0.0001 ^b^
Medial only	21	(12·3)	10	(6.1)		30	(17·7)	13	(7.9)	
Lateral only	18	(10·6)	11	(6.7)		15	(8·9)	2	(1.2)	
Corneal & peripheral*	11	(6·5)	6	(3.6)		14	(8·3)	8	(4.8)	
Medial & lateral	0	-	0	-		0	-	1	(0.6)	
Epilated	0	-	73	(44.2)		10	(5.9)	97	(58.8)	
***Trichiatic eyelash thickness***										
Normal thickness	89	(52·4)	37	(22.4)	<0.0001 ^b^	55	(32.5)	24	(14.6)	<0.0001 ^b^
Thinner	49	(28·8)	42	(25.5)		79	(46·7)	37	(22.4)	
Thicker	1	(0·6)	0	-		0	-	0	-	
Mixed lashes	31	(18·2)	13	(7.9)		25	(14·8)	7	(4.2)	
None (Epilated)	0	-	73	(44.2)		10	(5.9)	97	(58.8)	
***Trichiatic eyelash strength/stiffness***										
Normal strength	88	(51·8)	25	(15.2)	<0.0001 ^b^	50	(29·6)	14	(8.5)	<0.0001 ^b^
Weaker	50	(29·4)	56	(33.9)		84	(49·7)	48	(29.1)	
Stronger/stiffer	1	(0·6)	0	-		0	-	0	-	
Mixed lashes	31	(18·2)	11	(6.7)		25	(14·8)	6	(3.6)	
None (Epilated)	0	-	73	(44.2)		10	(5.9)	97	(58.8)	
***Trichiatic eyelash length***										
Normal length	42	(24·7)	12	(7.3)	<0.0001 ^b^	23	(13·6)	8	(4.8)	<0.0001^b^
Half length	54	(31·8)	33	(20.0)		50	(29·6)	28	(17.0)	
Shorter	32	(18·8)	24	(14.6)		60	(35.5)	19	(11.5)	
Mixed lashes	42	(24·7)	23	(13.9)		26	(15·4)	13	(7.9)	
None (Epilated)	0	-	73	(44.2)		10	(5.9)	97	(58.8)	
***Entropion grade*** ^c^										
None	8	(4·7)				62	(36·7)			
Mild	147	(86·5)				104	(61·5)			
Moderate	15	(8·8)				3	(1·8)			
Severe	0	-				0	-			

^a^ P-value from Poisson Regression

^b^ P-values for all categorical variables are from McNemar-Bowker test of symmetry just indicating the shift in a clinical characteristic between baseline and 6-month after epilation. Here each case is a match of its own before (baseline) and after (6 month) the measurement.

^c^ Mild = eyelid inward rotation without globe-lash base contact; Moderate = <50% eyelid inward rotation with globe-lash base contact; Sever = ≥50% of eyelid inward rotation with globe-lash base contact. Entropion data at follow-up is not needed in this study context as, a) epilation would not have effect on entropion, and b) disease progression that may result entropion grading change would not be observed within 6-months.

*Peripheral = trichiatic eyelashes not touching the cornea (but touching the eyeball medial or lateral to the cornea).

**Table 4:** Change in post-epilation trichiatic eyelashes burden and phenotype in unoperated TT cases (N = number of eyelashes touching the eye).

Characteristics	Baseline (N = 249)		1-month (N = 111)		2-month (N = 135)		3-month (N = 107)		4-month (N = 101)		5-month (N = 111)		6-month (N = 120)	
	n	(%)	n	(%)	n	(%)	n	(%)	n	(%)	n	(%)	n	(%)
***Trichiatic eyelash number*, *Mean (***95% CI)*	1.79	(1.62–1.97)	0.81	(0.60–1.02)	0.99	(0.80–1.19)	0.79	(0.61–0.97)	0.74	(0.57–0.91)	0.83	(0.66–1.01)	0.89	(0.71–1.08)
Mean difference (95% CI), vs baseline			-0.98	(-1.19– -0.77)	-0.81	(-1.02– -0.60)	-1.00	(-1.21– -0.80)	-1.05	(-1.25– - 0.84)	-0.97	(-1.17– -0.75)	-0.90	(-1.11– -0.69)
Trichiatic eyelash location*														
Corneal	192	(77.1)	74	(66.7)	102	(75.6)	67	(62.6)	82	(81.2)	80	(72.1)	86	(71.7)
OR (95% CI), vs baseline			0.59	(0.36–0.97)	0.92	(0.56–1.50)	0.50	(0.30–0.81)	1.28	(0.72–2.29)	0.77	(0.46–1.27)	0.75	(0.46–1.23)
Medial	33	(13.3)	18	(16.2)	16	(11.8)	25	(23.4)	12	(11.9)	17	(15.3)	22	(18.3)
OR (95% CI), vs baseline			1.27	(0.68–2.36)	0.88	(0.46–1.67)	2.00	(1.12–3.56)	0.88	(0.44–1.79)	1.18	(0.63–2.23)	1.47	(0.81–2.65)
Lateral	24	(9.6)	19	(17.1)	17	(12.6)	15	(14.0)	7	(6.9)	14	(12.6)	12	(10.0)
OR (95% CI), vs baseline			1.94	(1.01–3.70)	1.35	(0.70–2.61)	1.53	(0.77–3.04)	0.70	(0.29–1.68)	1.35	(0.67–2.73)	1.04	(0.50–2.16)
Trichiatic eyelash thickness*														
Normal thickness	146	(58.6)	36	(32.4)	52	(38.5)	39	(36.4)	34	(33.7)	47	(42.3)	53	(44.2)
OR (95% CI), vs baseline			0.34	(0.21–0.55)	0.44	(0.29–0.68)	0.40	(0.25–0.63)	0.36	(0.22–0.58)	0.52	(0.33–0.81)	0.56	(0.36–0.87)
Thinner than normal	100	(40.2)	75	(67.6)	83	(61.5)	68	(63.6)	67	(66.3)	62	(55.9)	67	(55.8)
OR (95% CI), vs baseline			3.10	(1.94–4.97)	2.38	(1.55–3.65)	2.60	(1.63–4.15)	2.94	(1.81–4.77)	1.89	(1.20–2.96)	1.88	(1.21–2.93)
Thicker than normal	3	(1.2)	0	(0.0)	0	(0.0)	0	(0.0)	0	(0.0)	2	(1.8)	0	(0.0)
OR (95% CI), vs baseline			-	-	-	-	-	-	-	-	1.50	(0.25–9.13)	-	-
Trichiatic eyelash strength*														
Normal strength	147	(59.0)	33	(29.7)	49	(36.3)	35	(32.7)	20	(19.8)	34	(30.6)	35	(29.2)
OR (95% CI), vs baseline			0.29	(0.18–0.47)	0.39	(0.26–0.61)	0.34	(0.21–0.54)	0.17	(0.10–0.30)	0.31	(0.19–0.49)	0.29	(0.18–0.46)
Weaker contact	99	(39.8)	78	(70.3)	86	(63.7)	72	(67.3)	81	(80.2)	75	(67.6)	85	(70.8)
OR (95% CI), vs baseline			3.58	(2.22–5.79)	2.66	(1.72–4.10)	3.12	(1.93–5.02)	6.14	(3.54–10.6)	3.16	(1.97–5.06)	3.68	(2.30–5.88)
Stronger contact	3	(1.2)	0	(0.0)	0	(0.0)	0	(0.0)	0	(0.0)	2	(1.8)	0	(0.0)
OR (95% CI), vs baseline			-	-	-	-	-	-	-	-	1.50	(0.25–9.13)	-	-
Trichiatic eyelash length*														
Normal length	94	(37.8)	22	(19.8)	37	(27.4)	9	(8.4)	12	(11.9)	19	(17.1)	25	(20.8)
OR (95% CI), vs baseline			0.41	(0.24–0.70)	0.62	(0.39–0.98)	0.15	(0.07–0.31)	0.22	(0.11–0.43)	0.34	(0.19–0.59)	0.43	(0.26–0.72)
Half length	77	(30.9)	47	(42.3)	69	(51.1)	67	(62.6)	45	(44.5)	53	(47.7)	52	(43.3)
OR (95% CI), vs baseline			1.64	(1.03–2.61)	2.33	(1.52–3.59)	3.74	(2.33–6.02)	1.79	(1.12–2.89)	2.04	(1.29–3.23)	1.71	(1.09–2.68)
Short	78	(31.3)	42	(37.8)	29	(21.5)	31	(29.0)	44	(43.6)	39	(35.1)	43	(35.8)
OR (95% CI), vs baseline			1.33	(0.84–2.13)	0.60	(0.37–0.98)	0.89	(0.54–1.47)	1.69	(1.05–2.72)	1.19	(0.74–1.90)	1.22	(0.77–1.94)
***Trichiatic eyelashes epilated by examiner***^***+***^														
Total (% out of in 6-month) ^a^	313	(24.3)	156	(12.1)	189	(14.7)	146	(11.3)	150	(11.6)	165	(12.8)	169	(13.1)
Person level, mean (SD)	1.84	(1.09)	0.93	(1.38)	1.13	(1.26)	0.88	(1.10)	0.90	(1.19)	1.01	(1.23)	1.03	(1.35)
***Trichiatic eyelash epilated by patients***^***+***^														
Total (% out of in 6-month) ^b^	-	(-)	16	(17.8)	25	(27.8)	12	(13.3)	18	(20.0)	5	(5.6)	14	(15.6)
Person level, mean (SD)	-	(-)	0.10	(0.57)	0.15	(0.65)	0.07	(0.50)	0.11	(0.60)	0.03	(0.26)	0.08	(0.45)
***Number of cases epilating*, *n/N (%)*** ^***c***^	31/170	(18.2)	5/168	(3.0)	10/168	(6.0%)	5/167	(3.0)	6/167	(3.6)	3/165	(1.8)	6/165	(3.6)

Notes: Analyses are done on trichiatic eyelashes (not cases), unless specified.

* Analysis done excluding prior-epilators using generalised linear binomial regression model, except the trichiatic eyelash burden data, where random effect linear regression model was used. The ORs and their 95% CIs indicate the odds of a post-epilation trichiatic eyelash phenotype change between baseline and the different follow-up time points. For instance, for the trichiatic eyelash location data the ORs and the 95% CIs for the corneal eyelashes show how much the odds of a trichiatic eyelash being corneal among all trichiatic eyelashes at each follow-up time point compared to baseline.

^**+**^Analysis done on the trichiatic eyelashes of all unoperated TT cases.

^a^ Denominator is the total number of trichiatic eyelashes epilated at baseline and the 6-month follow-up period by the examiner = 1288 eyelashes.

^b^ Denominator is the total number of eyelashes epilated by the patient at home (reported by the patient) in the 6-month follow-up period = 90 eyelashes.

^c^ Analysis done on study participants.

#### Postoperative TT Cases

Six-month after frequent epilation the majority (168/169) of postoperative TT cases remained minor (≤5 eyelashes touching the eye or evidence of epilation of <1/3^rd^ of the upper eyelid), 1 person (0.6%) had >5 eyelashes touching the eye, Table 3. There were 23/169 (13.6%) postoperative TT cases with no sign of trichiasis (had neither eyelash touching the eye nor epilation) after the baseline epilation. These cases at baseline had less trichiatic eyelash burden (mean lash burden [SD] 1.0 [0.5] vs 1.8 [1.3]; Wilcoxon rank-sum test p = 0.0019). In addition, higher proportions of the postoperative TT cases with no sign of trichiasis tended to have peripheral only trichiatic eyelashes (touching the eye either medial or lateral to the cornea; 8/23 [34.8%] vs 37/146 [25.3%]; Chi square p = 0.34), and misdirected (3/23 [13.0%] vs 7/14 [4.8%]; Chi square p = 0.17) only trichiatic eyelashes than those with sign of trichiasis at 6-month. The mean number of trichiatic eyelashes and phenotype changes between baseline and the different follow-up time points are shown in Table 5. There was evidence of a reduction in trichiatic eyelash burden in postoperative TT cases between baseline and 6-month: mean lash difference = -1.16 (-1.36– -0.95); Poisson regression, RR = 0.34 (95% CI, 0.26–0.45); p<0.0001. There was evidence that post-epilation PTT eyelashes at 6-month had higher odds of being weak (p = 0.039), and half-length (p = 0.013) than the pre-epilation trichiatic eyelashes at baseline, Table 5. There was no evidence of a change in PTT eyelashes location and thickness between baseline and at the 6-month follow-up. There was no evidence of a difference in trichiatic eyelashes phenotypes between prior-epilators (n = 52) and non-prior-epilators in postoperative TT cases at baseline (data not provided). At baseline, 122 (72%) PTT cases had only aberrant trichiatic eyelashes (111 [65.7%] had metaplastic, and 11 [6.5%] had misdirected trichiatic eyelashes) (S1 Fig).

**Table 5:** Change in post-epilation trichiatic eyelashes phenotype in postoperative TT cases (N = number of eyelashes touching the eye).

Characteristics	Baseline (N = 205)		1-month (N = 88)		2-month (N = 103)		3-month (N = 85)		4-month (N = 83)		5-month (N = 98)		6-month (N = 69)	
	n	(%)	n	(%)	n	(%)	n	(%)	n	(%)	n	(%)	n	(%)
*Trichiatic eyelash number*, mean (95% CI)*	1.75	(1.53–1.97)	0.75	(0.55–0.95)	0.89	(0.68–1.09)	0.73	(0.54–0.92)	0.72	(0.55–0.88)	0.86	(0.66–1.06)	0.60	(0.43–0.77)
Mean difference (95% CI), vs baseline			-1.00	(-1.20– - 0.80)	-0.87	(-1.07– -0.67)	-1.02	(-1.22– -0.82)	-1.04	(-1.24– - 0.84)	-0.90	(-1.11– -0.70)	-1.16	(-1.36– -0.95)
Trichiatic eyelash location*														
Corneal	128	(62.4)	54	(61.4)	56	(54.4)	42	(49.4)	53	(63.9)	64	(65.3)	41	(59.4)
OR (95% CI), vs baseline			0.96	(0.57–1.60)	0.72	(0.44–1.6)	0.59	(0.35–0.97)	1.06	(0.63–1.80)	1.13	(0.68–1.87)	0.88	(0.50–1.54)
Medial	49	(23.9)	22	(25.0)	26	(25.2)	29	(34.1)	25	(30.1)	23	(23.5)	23	(33.3)
OR (95% CI), vs baseline			1.06	(0.59–1.89)	1.07	(0.62–1.86)	1.65	(0.95–2.86)	1.37	(0.78–2.42)	0.98	(0.55–1.72)	1.59	(0.88–2.88)
Lateral	28	(13.7)	12	(13.6)	21	(20.4)	14	(16.5)	5	(6.0)	11	(11.2)	5	(7.2)
OR (95% CI), vs baseline			1.00	(0.48–2.07)	1.62	(0.87–3.02)	1.25	(0.62–2.51)	0.40	(0.15–1.09)	0.80	(0.38–1.68)	0.49	(0.18–1.33)
Trichiatic eyelash thickness*														
Normal thickness	75	(36.6)	19	(21.6)	20	(19.4)	21	(24.7)	26	(31.3)	27	(27.6)	22	(31.9)
OR (95% CI), vs baseline			0.48	(0.27–0.85)	0.42	(0.24–0.73)	0.57	(0.32–1.00)	0.79	(0.46–1.36)	0.66	(0.39–1.12)	0.81	(0.45–1.45)
Thinner than normal	130	(63.4)	69	(78.4)	83	(80.6)	64	(75.3)	57	(68.7)	71	(72.4)	47	(68.1)
OR (95% CI), vs baseline			2.10	(1.17–3.75)	2.39	(1.36–4.21)	1.76	(0.99–3.11)	1.26	(0.73–2.18)	1.52	(0.90–2.57)	1.23	(0.69–2.20)
Thicker than normal	0	(0.0)	0	(0.0)	0	(0.0)	0	(0.0)	0	(0.0)	0	(0.0)	0	(0.0)
OR (95% CI), vs baseline			-	-	-	-	-	-	-	-	-	-	-	-
Trichiatic eyelash strength*														
Normal strength	69	(33.7)	12	(13.6)	14	(13.6)	10	(11.8)	10	(12.0)	18	18.4)	14	(20.3)
OR (95% CI), vs baseline			0.31	(0.16–0.61)	0.31	(0.16–0.58)	0.26	(0.13–0.54)	0.27	(0.13–0.56)	0.44	(0.25–0.80)	0.50	(0.26–0.96)
Weaker contact	136	(66.3)	76	(86.4)	89	(86.4)	75	(88.2)	73	(88.0)	80	(81.6)	55	(79.7)
OR (95% CI), vs baseline			3.21	(1.64–6.31)	3.22	(1.71–6.08)	3.80	(1.85–7.82)	3.70	(1.80–7.62)	2.25	(1.25–4.06)	1.99	(1.03–3.83)
Stronger contact	0	(0.0)	0	(0.0)	0	(0.0)	0	(0.0)	0	(0.0)	0	(0.0)	0	(0.0)
OR (95% CI), vs baseline			-	-	-	-	-	-	-	-	-	-	-	-
Trichiatic eyelash length*														
Normal length	34	(16.6)	10	(11.4)	7	(6.8)	7	(8.2)	6	(7.2)	14	(14.3)	6	(8.7)
OR (95% CI), vs baseline			0.64	(0.30–1.37)	0.37	(0.16–0.86)	0.45	(0.19–1.06)	0.39	(0.16–0.97)	0.84	(0.43–1.65)	0.48	(0.19–1.20)
Half length	65	(31.7)	26	(29.5)	49	(47.6)	41	(48.2)	39	(47.0)	36	(36.7)	37	(53.6)
OR (95% CI), vs baseline			0.90	(0.52–1.56)	1.95	(1.20–3.18)	2.01	(1.20–3.37)	1.91	(1.13–3.22)	1.25	(0.75–2.07)	2.49	(1.49–4.35)
Short	106	(51.7)	52	(59.1)	47	(45.6)	37	(43.5)	38	(45.8)	48	(49.0)	26	(37.7)
OR (95% CI), vs baseline			1.35	(0.81–2.24)	0.78	(0.49–1.26)	0.72	(0.43–1.20)	0.79	(0.47–1.31)	0.90	(0.55–1.45)	0.56	(0.32–0.99)
***Trichiatic eyelash epilated by examiner***^***+***^														
Total (% out of in 6-month) ^a^	286	(26.0)	137	(12.4)	158	(14.4)	128	(11.6)	130	(11.8)	153	(13.9)	108	(9.8)
Person level, mean (SD)	1.69	(1.22)	0.81	(1.19)	0.94	(1.14)	0.77	(1.10)	0.78	(0.94)	0.93	(1.29)	0.65	(0.99)
***Trichiatic eyelash epilated by patient***^***+***^														
Total (% out of in 6-month) ^b^	-	(-)	32	(18.4)	33	(18.9)	18	(10.3)	33	(18.9)	32	(18.4)	26	(14.9)
Person level, mean (SD)	-	(-)	0.19	(0.73)	0.20	(0.73)	0.11	(0.50)	0.20	(0.75)	0.19	(0.80)	0.16	(0.67)
***Number of cases epilating*, *n/N (%)*** ^***c***^	52/169	(30.8)	13/168)	(7.7)	12/168	(7.1)	8/167	(4.8)	13/167	(7.8)	11/165	(6.7)	10/165	(6.1)

NB: Analyses are done on trichiatic eyelashes (not cases), unless specified.

* Analysis done excluding prior-epilators using generalised linear binomial regression model, except the trichiatic eyelash burden data, where random effect linear regression model was used. The ORs and their 95% CIs indicate the odds of a post-epilation trichiatic eyelash phenotype change between baseline and the different follow-up time points. For instance, for the trichiatic eyelash location data the ORs and the 95% CIs for the corneal eyelashes show how much the odds of a trichiatic eyelash being corneal among all trichiatic eyelashes at each follow-up time point compared to baseline.

^**+**^Analysis done on the trichiatic eyelashes of all postoperative TT cases.

^a^ Denominator is the total number of trichiatic eyelashes epilated at baseline and in the 6-month follow-up period by the examiner = 1100 eyelashes.

^b^ Denominator is the total number of eyelashes epilated by the patient at home (reported by the patient) in the 6-month follow-up period = 174 eyelashes.

^c^ Analysis done on study participants.

#### Unoperated and Postoperative TT Cases Comparisons

At baseline, there was no significant difference in the total number of trichiatic eyelashes between postoperative (169) and unoperated TT (170) cases (total trichiatic eyelashes 286 vs 313, RR = 0.92, [95% CI, 0.78–1.08]; p = 0.31). However, postoperative cases had a higher proportion of medial (64 [22.4%] vs 36 [11.5%], RR = 1.89, [95% CI, 1.25–2.85]; p = 0.0025), non-entropic (80 [27.9%] vs 40 [12.8%], RR = 2.13, [95% CI, 1.46–3.13]; p = 0.0001), thin (181 [63.3%] vs 125 [39.9%], RR = 1.46, [95% CI, 1.16–1.84]; p = 0.0012), weak (187 [65.4%] vs 124 [39.6%], RR = 1.53, [95% CI, 1.22–1.92]; p = 0.0030), and short (147 [51.4%] vs 105 [33.5%], RR = 1.39, [95% CI, 1.08–1.80]; p = 0.010) trichiatic eyelashes than the unoperated cases. In the six-month follow-up period, the postoperative cases had significantly lower burden of post-epilation trichiatic eyelashes than the unoperated cases (total post-epilation trichiatic eyelashes: 814 vs 978, RR = 0.79, [95% CI, 0.72–0.86]; p<0.0001).

### Surgical Management Willingness

#### Present willingness

Of all study participants, 5/169 (3.0%) in the unoperated and 4/169 (2.4%) in the post-operative group expressed willingness to undergo surgery during the 6 follow-up timepoints, and were referred for surgery, of which 2 (1 from each group) had been operated during the 6-month follow-up. The five unoperated cases who wanted to undergo surgery tended to have lower trichiatic eyelash burden than those who didn’t want, both at baseline (mean lash burden [SD]: 1.4[0.9] vs 1.9 [1.1]), and 6-month follow-up (mean lash burden [SD]: 0.2 [0.4] vs 1.1 [1.3]). On the other hand, the 4 postoperative cases who wanted to undergo repeat surgery tended to have higher burden of trichiatic eyelashes than those who didn’t want both at baseline (mean lash burden [SD]: 2.5 [1.9] vs 1.7 [1.2]), and 6 month (mean lash burden [SD]: 1.0 [0] vs 0.6 [0.9]). There were no differences in other trichiasis phonotypes between those who wanted to have surgery and those who didn’t.

#### Future willingness

The majority of the unoperated (120/164 [73.2%]) and postoperative (134/163 [82.2%] TT cases consistently responded at all six follow-up time points that they would accept surgery if their trichiasis progressed. Awareness of the presence of trichiasis at baseline had no influence on TT surgery acceptance in the unoperated cases: among those accepted to receive surgical management if the TT progressed, 72/129 (55.8%) were aware and 57/75 (44.2%) were unaware that they had trichiasis (p = 0.97). In addition, there was no difference in willingness to accept surgical management at baseline between prior-epilators and non-prior-epilators in both the unoperated (83.9% vs 74.1%, p = 0.25) and postoperative (78.8% vs 73.5%, p = 0.46) TT groups.

### Corneal Opacity (CO) and Vision Changes

CO and vision change results between baseline and follow-ups are presented in Table 6. There was no evidence of progression in CO photo grading between baseline and 6-month follow-up in either unoperated or postoperative TT cases, although the field grading analysis indicated evidence of progression (OR = 1.80 [95% CI, 1.22–2.65]; p = 0.0030) in unoperated cases most of which (57.1%) occurred in the periphery (opacity not entering the central 4mm of the cornea). There was evidence of vision improvement at the 6-month follow-up in the unoperated group, while no similar change was seen in the postoperative group.

**Table 6:** Corneal opacity and vision changes between baseline and 6-month in unoperated and postoperative TT cases.

*Variables*	*Unoperated TT Cases*							*Postoperative TT Cases*						
	*Baseline* *N = 170*		*6-month* *N = 165*		*OR*	*95% CI*	*P-value*	*Baseline* *N = 169*		*6-month* *N = 165*		*OR*	*95% CI*	*P-value*
	*n*	*(%)*	*n*	*(%)*				*n*	*(%)*	*n*	*(%)*			
***Corneal opacity (CO)***														
None (CC0)	89 ^***a***^	(52·7)	83	(50.3)	1.05	(0.70–1.57)	0.80	51	(30·2)	50	(30.3)	1.00	(0.68–1.47)	0.98
Peripheral (CC1)*	26	(15·4)	30	(18.2)				30	(17·7)	29	(17.6)			
Off centre faint (CC2a)	22	(13·0)	21	(12.7)				27	(16·0)	26	(15.8)			
Off centre dense (CC2b)	1	(0·6)	1	(0.6)				4	(2·4)	4	(2.4)			
Central faint (CC2c)	28	(16·6)	27	(16.4)				47	(27·8)	46	(27.9)			
Central dense (CC2d)	2	(1·2)	2	(1.2)				8	(4·7)	8	(4.8)			
Total central dense (CC3)	0	-	0	-				2	(1.2)	2	(1.2)			
Phthisis (CC4)	1	(0.6)	1	(0.6)				0	-	0	-			
***Presenting logMAR VA in study eye***														
≥6/12 (Normal Vision)	80	(47.1)	110	(66.7)	0.48	(0.31–0.74)	0.0010	58	(34.3)	65	(39.4)	0.85	(0.57–1.26)	0.41
<6/12 - ≥6/18 (Mild VI)	23	(13·5)	11	(6.7)				24	(14·2)	22	(13.3)			
<6/18 - ≥6/60 (Moderate VI)	57	(33·5)	34	(20.6)				74	(43·8)	65	(39.4)			
<6/60 - ≥3/60 (Severe VI)	1	(0·6)	2	(1.2)				3	(1·8)	5	(3.0)			
<3/60 (Blind)	9	(5·3)	8	(4.8)				10	(5·9)	8	(4.8)			

Note: Odds ratios are from ordinal logistic regression. Corneal opacity is from photographic grading. ^**a**^ One missing value. The vision data analysis is adjusted for age.

*Opacity not entering the central 4mm of the cornea.

### Epilation practices

During the six-month period, 2,391 trichiatic eyelashes were removed by the examiner: 1,291 (54.0%) in the unoperated group and 1,100 (46.0%) in the postoperative group. Among these, 2,186 (91.4%) were completely epilated and 205 (8.6%) were incompletely epilated. More eyelashes in the postoperative (119 [58.0%]) than the unoperated group (86 [41.9%) were incompletely epilated (OR = 1.41 [95% CI 1.03–1.91]; p = 0.030). Mild pain was felt by 18/168 (10.7%) unoperated, and 3/169 (1.78%) postoperative cases (p = 0.001) during examiner epilation. Among those epilating at home using traditional forceps, 7/20 (35.0%) unoperated and 7/29 (24.1%) postoperative TT cases (p = 0.41) reported pain (any severity).

After adjusting for the number of eyelashes touching the eye, the overall number of cases who epilated at home during the six follow-up timepoints was significantly lower than the number epilating at home at baseline in both the unoperated group (OR = 0.71 [95% CI 0.62–0.82]; p<0.0001) and postoperative group (OR = 0.71 [95% CI 0.64–0.79]; p<0.0001) (actual data provided in Tables 4 and 5). However, there was no variation on the number of cases epilating at home between the six follow-up timepoints (1 to 6-month) in both groups (OR = 0.95 [95% CI 0.85–1.07]; p = 0.43). There was no difference in the proportion epilating at home between the unoperated (20/169 [11.8%]) and postoperative (29/169 [17.2%]) groups, (p = 0.16). However, among those epilating at home, postoperative TT cases tended to epilate more frequently than the unoperated TT cases in the 6-month follow-up period (Mean frequency [SD] 1.20 [0.41] vs 1.45 [1.12], p = 0.094), epilating a total number of 90 and 174 eyelashes respectively. There was no difference in the median (IQR) number of home-epilated eyelashes per participant between the unoperated TT: 4 (2–8), and the postoperative TT: 4 (2–9) (p = 0.15) cases. Participants who were aware of having trichiasis at baseline were more likely to epilate at home during the 6-month follow-up period than those not aware: UTT, 20.2% vs 1.3% (OR = 18.5 [95% CI, 2.41–141.7]; p = 0.0050); and PTT, 24.1% vs 1.9%, (OR = 16.5 [95% CI, 2.19–125.2]; p = 0.0066).

### Vision related quality of life changes

The VRQoL of cases were measured at baseline, one month and six-months. VRQoL scores of both unoperated and postoperative TT cases increased substantially in all four subscales both at 1-month and 6-months (Table 7). The largest improvements were seen in the visual symptom sub-scale in both groups at both follow-up time points.

**Table 7:** Vision related quality of life (VRQoL) change between baseline and follow-up time points in unoperated and postoperative TT cases.

Domains	Baseline (N = 168)		1-month (N = 168)		Baseline vs 1-month			Baseline (N = 165)		6-month (N = 165)		Baseline vs 6-month		
	*Mean*	*(95% CI)*	*Mean*	*(95% CI)*	*Difference*	*(95% CI)*	*P-value*	*Mean*	*(95% CI)*	*Mean*	*(95% CI)*	*Difference*	*(95% CI)*	*P-value*
Overall eyesight														
UTT Cases	50.9	(47.7–54.1)	59.8	(56.9–62.7)	8.9	(5.2–12.7)	<0.0001	50.8	(47.6–53.9)	70.8	(67.7–73.7)	20.0	(16.5–23.5)	<0.0001
PTT Cases	50.4	(47.3–53.6)	58.5	(55.7–61.2)	8.0	(4.5–11.6)	<0.0001	50.6	(47.4–53.8)	68.6	(65.5–71.8)	18.0	(14.7–21.4)	<0.0001
Visual symptom														
UTT Cases	59.7	(56.0–63.3)	86.2	(83.4–88.9)	26.5	(22.9–30.1)	<0.0001	59.5	(55.8–63.2)	86.9	(84.3–89.5)	27.4	(24.0–30.7)	<0.0001
PTT Cases	61.2	(57.6–64.9)	85.8	(82.9–88.7)	24.6	(21.0–28.2)	<0.0001	61.3	(57.6–65.0)	83.6	(80.8–86.5)	22.4	(19.3–25.5)	<0.0001
General functioning														
UTT Cases	84.7	(82.0–87.4)	92.8	(90.8–94.8)	8.1	(5.7–10.4)	<0.0001	84.7	(82.0–87.4)	94.8	(93.0–96.7)	10.1	(7.9–12.3)	<0.0001
PTT Cases	78.8	(75.4–82.3)	89.9	(87.4–92.5)	11.1	(9.0–15.9)	<0.0001	79.1	(75.7–82.6)	91.5	(89.0–93.5)	12.3	(9.8–14.9)	<0.0001
Psychosocial														
UTT Cases	75.3	(71.7–78.9)	87.8	(84.7–90.9)	12.4	(8.3–14.7)	<0.0001	75.4	(71.9–79.0)	86.8	(83.9–89.8)	11.4	(8.4–14.4)	<0.0001
PTT Cases	70.6	(66.5–74.8)	85.7	(82.1–89.3)	15.1	(11.4–18.7)	<0.0001	70.7	(66.5–74.9)	84.7	(81.6–87.7)	13.9	(10.7–17.2)	<0.0001

Note: Mean and mean differences are generated from paired t-test; p-values are from a random effect linear regression model adjusted for age, gender, self-rated wealth and best eye presenting vision of the person at each time point.

## Discussion

There has been a significant reduction in the estimated global TT backlog due to the unprecedented effort to avert the risk of blindness through corrective eyelid surgery. However, a reduction in TT surgery uptake in the remaining unoperated cases (Carter Center Program Review, 2018) and the number of people managed for TT globally has been reported.[26] Among many factors, the key is likely the large proportion of the remaining backlog of cases are cases with just a few trichiatic eyelashes who either are not aware of having the trichiasis or do not wish to have surgery. Promoting surgery for all unoperated and postoperative TT cases is unlikely to have a significant impact on the backlog if the patients do not want to be operated, and an alternative management strategy is urgently needed.

Despite a strong trachoma control programme with robust community-based screening and surgical services, nearly half of the unoperated and about a third of the postoperative TT cases enrolled into this study were unaware that they had trichiasis. Minor trichiasis (≤5 eyelashes touching the eye or evidence of epilation of <1/3^rd^ of the upper eyelid) is less likely to be painful and visually troubling than major trichiasis (>5 eyelashes touching the eye or evidence of epilation of ≥1/3^rd^ of the upper eyelid). More than half of the cases had only a single lash touching the eye, which are likely to be missed during screening and impact and surveillance surveys. These data suggest that, firstly, there are still a significant proportion of people with minor TT who are unknown to the health system, a situation likely to persist well beyond 2020. Secondly, surgery is probably a less appealing option for such cases, who do not have both awareness of the trichiasis and a ‘felt need’ for intervention. This is possibly why half of the unoperated and postoperative TT cases in this study considered the less invasive epilation to be an adequate treatment alternative for their trichiasis. Other studies also reported that not having symptom from the trichiasis and symptoms being not bad enough are among barriers for surgical uptake.[5,27] Epilation would give such patients adequate time to look into their condition and other possible management options.

The evidence from this study indicates that frequent epilation helps to successfully control and reduce the burden of trichiasis in minor cases. The number of post-epilation trichiatic eyelashes was significantly reduced at all follow-up time points by more than half with only 3 cases progressing to >5 eyelashes touching the eye. These results are similar to a clinical trial results that showed only 13% of minor unoperated TT cases progressed to major trichiasis in a two-year period; and among those who kept epilating for four years, 54.1% had no eyelashes touching the eye and only 2.6% progressed to major trichiasis.[9,14] The change in the reduction of eyelash burden would range from 5 to 4 (a 20% reduction) and 1 to 0 (a 100% reduction). Although there is no clear evidence to support it, a reduction of trichiasis burden by one eyelash would probably have higher clinical significance in minor TT cases than those with major TT. In our study, at baseline only a handful of our study participants from the unoperated (3.5%) and the postoperative (5.3%) groups had 5 trichiatic eyelashes. Otherwise about 50% in both groups had 1 trichiatic eyelash and about 20% had 2 trichiatic eyelashes in which a single eyelash reduction means a 100% and a 50% reduction respectively. This will have a huge clinical significance to the patients and underpins the whole purpose of epilation—mitigating the pain and the risk of vision impairment until a surgical option is viable. A study reported that eyes with evidence of epilation but still with some eyelashes touching the eye (incomplete epilation) were less likely to have CO than eyes with trichiasis but with no evidence of epilation suggesting that some amount of epilation is clinically meaningful even if it is not complete enough.[19]

Some cases from both groups had neither any eyelash touching the eye nor epilated in the 6-month follow-up period. Our limited data indicates that these cases at baseline tended to have fewer, peripheral and misdirected trichiatic eyelashes indicating that, in such cases, a single epilation would probably be sufficient to treat completely. Similar results have been reported in a longitudinal study conducted in the Gambia where 5% and 7% of unoperated major TT cases no longer had signs of trichiasis after 6 and 12 months respectively.[3] This same study showed that subjects with minor TT and treated with epilation at baseline were more likely to be free of signs of trichiasis at 6 and 12 months follow-ups than the unoperated major TT cases.[3]

Post-epilation trichiatic eyelashes were more likely to be weak, thin and short or half-length than pre-epilation trichiatic eyelashes. A possible explanation is that the post-epilation trichiatic eyelashes might not have completed growing at the time of assessment. However, the growth phase (anagen) of a new eyelash would take about 1–2 months [28], while incompletely epilated eyelashes would probably take a shorter period to grow back, suggesting that the monthly follow-up employed in this study would allow to capture fully grown post-epilation lashes. In addition, the phenotype of the trichiatic eyelashes of people already epilating for some time prior to enrolment, which would have a fully-grown post-epilation trichiatic eyelashes, was similar to that observed in people who just started epilation after enrolment. In current practice, trichiasis is diagnosed and treated if an eyelash touches the eye, not based on its length or thickness. It would be unethical to wait until a trichiatic eyelash fully grows to observe or manage with epilation or surgery. It is also possible that the more troubling thicker, stronger, and longer trichiatic eyelashes are being epilated by the patients leaving a higher number of thin, weak and short trichiatic eyelashes which pose lesser risk. This is aligned with the whole point of epilation and underpins the need for frequent practice.

Direct comparison of photographs indicated that there is no change in CO grading between baseline and at the 6-month follow-up in both the unoperated and postoperative TT groups, while the field grading in the unoperated group showed mostly a peripheral progression (not involving the central 4mm of the cornea). This is in line with the finding that post-epilation trichiatic eyelashes were more likely to be shorter than the pre-epilation trichiatic eyelashes, and hence be less likely to grow sufficiently long to reach the centre of the cornea. This is similar to the result of a clinical trial we conducted a few years ago in which photographic CO grading showed no change in minor unoperated cases recruited in the same study setting and randomised to the epilation arm while the field grading indicated a moderate variation.[9] We have previously discussed in detail why differences between photographic and field grading might occur and our preference for direct comparison of magnified high resolution photographs over field grading as a reliable measure of CO change.[9] There was vision improvement in the unoperated cases 6-month after epilation, but not in the postoperative who tended to be older and had more corneal opacity at baseline. Old age and presence of more corneal opacity were negatively associated with vision improvement in other studies.[4,14]

Epilation significantly improved VRQoL regardless of vision change for both unoperated and postoperative TT cases. The largest improvements were seen in the visual symptom subscale in both groups indicating that epilation is capable of relieving pain and discomfort from the trichiasis. Although, the effect was smaller, the VRQoL finding in this study is similar to what has been reported for TT surgery in earlier studies.[22,29,30] The change in overall eyesight was greater at the 6-month follow-up than the 1-month, suggesting that frequent epilation would have a greater effect than a one-time epilation in improving the patients visual perception. The improvement in VRQoL may have contributed to more than 97% of cases declining surgical management in the six-month period. However, this might have also been influenced by the fact that these study participants are regularly assisted with epilation by the examiner. It is possible that these results would be different in people who have to perform all their epilation at home. About three quarters of the cases consistently expressed their willingness to accept surgery if epilation failed to control the trichiasis suggesting that the programmatic implementation of epilation at least in this study population would not hamper surgical acceptance. However, these results may not be generalisable to other settings as surgical acceptance and quality of life can be influenced by several factors.

This is the first longitudinal study with detailed phenotyping of post-epilation trichiatic eyelashes and measuring the effect of epilation in managing postoperative TT cases and its impact on quality of life. We employed logistically intensive frequent follow-ups with high follow-up rates to capture fast growing post-epilation trichiatic eyelashes. The interviews and examinations were conducted by the same persons at each visit, increasing the reliability of the data presented. However, the study has a number of limitations. The frequent follow-up and case management employed in this study may not reflect the realities in programmatic settings. There are no comparison participants that could have helped to explain what would have happened to people without trichiasis, and to people with trichiasis but who refused both epilation and surgery to support the trichiatic eyelash burden and phenotype, corneal opacity, vision, and quality of life change results. Post-epilation eyelash phenotype was studied only for the eyelashes touching the eye. It is possible that the characteristics of some post-epilation eyelashes pointing towards the eye but yet to touch it would be different. However, our purpose was to study post-epilation “trichiatic eyelashes”, and an eyelash not touching the eye is not a trichiatic eyelash. Importantly eyelashes not touching the eye were not epilated at any follow-up time point, therefore would not be captured in any of the next follow-up when they touch the eye given that not many patients were frequently epilating at home. The gradings have been conducted by a single examiner and quality assurance performed within the study team. Although we used a clear grading system eyelash strength and thickness are not always independent of each other. We recognise photo grading on the eyelashes phenotypes by an independent expert would have strengthened the results. The participants were advised to epilate at home after a training on how to epilate but only some practiced home epilation during the follow-up period. However, they were not provided epilation forceps as this was not permitted by the Ministry of Health of Ethiopia. Thus, most cases might have preferred to wait until the next follow-up for their eyes to be epilated by the study team with machine made forceps than by a community member with traditional forceps. This is evident from the fact that the proportion of cases epilating at home have significantly reduced during the follow-up period compared to the baseline. The small frequencies of homebased-epilation could not allow detailed analysis and limited the ability of the study in measuring its impact versus health facility-based epilation. However, in a previous trial that provided training to a friend or relative to do the epilation and two pairs of epilation forceps, we found results comparable with those in the present study mainly on trichiatic eyelash burden reduction and CO changes.[9,14] Surgical management willingness data was collected using structured questionnaire at baseline and at each follow-up time point. A qualitative component to explore why most participants preferred epilation over surgery would have enriched the results and could be considered as a follow-up study.

Overall, we found evidence that frequent epilation neither increases the burden of trichiasis nor results in more damaging stiff and thick post-epilation trichiatic eyelashes. In this study setting, cases with minor TT reported a willingness to accept corrective surgery if their trichiasis progressed, potentially contradicting the assumption that the programmatic implementation of epilation would affect surgical uptake. WHO in its Second Global Scientific Meeting on TT, recommended that “It is ethically important to inform the patient with minor trichiasis about both surgery and epilation, and to discuss the benefits and risks of each procedure so that an informed decision can be made”.[15] Patients with few trichiatic eyelashes should not receive possibly unnecessary, and potentially disfiguring operation while there is a less invasive alternative management with satisfactory outcome that could be implemented until and only if surgical management is required. National programmes may consider epilation as a second line alternative to surgery for unoperated TT cases with trichiatic eyelashes touching the cornea and can be offered to such cases who decline surgery. In addition, epilation can be provided as a first line option of management along with surgery for minor postoperative TT cases and, unoperated TT cases with peripheral trichiasis and with no or mild entropion, so that the patients can choose between the two management strategies. The programmatic implementation of epilation should involve: a) training the patients and a close family member on how to successfully epilate without breaking lashes; b) provision of machine made epilation forceps with regular follow-up to ensure that they are epilating properly and examine any sign of progression and offer surgery as per clinical and patient needs; and c) effective recording and the removal of epilation treated cases from the backlog of TT cases unknown to the health system to avoid recycling. The programmatic implementation of epilation would help to address the needs of millions of TT cases constituting more than half of the global backlog and thereby achieve the global elimination targets. Additional research further investigating the implications of programme wide implementation of epilation on the community and the surgical services in various settings would enrich the evidence base.

## Supporting information

S1 Table Secondary analysis of data on postoperative trachomatous trichiasis (PTT) severity from two randomised control trials [1,2] conducted in the same study area as this study. (PDF)

S1 Fig Eyelids with Postoperative Trachomatous Trichiasis (PTT). (TIFF)
