# Supplementary material for: Effect of repeated epilation for minor trachomatous trichiasis on lash burden, phenotype and surgical management willingness: A cohort study
Source: PLoS Negl Trop Dis. 2020 Dec 14;14(12):e0008882. doi: 10.1371/journal.pntd.0008882 (PMC7769600; doi:10.1371/journal.pntd.0008882)
Supplement: S1 Table — (PDF) [file pntd.0008882.s001.pdf]

**S1 Table: Secondary analysis of data on postoperative trachomatous trichiasis (PTT) severity from two randomised control trials [1, 2] conducted in the same study area as this study.**

This table shows the majority of the cases which developed postoperative trichiasis 12 months after corrective eyelid surgery had little or no entropion (~90%), had aberrant (~70%) and few (one or two eyelashes [~60%]) touching the eye.

| Characteristic | PLTR vs BLTR Trial N=131 | | Doxycycline Trial N=91 | |
|---|---|---|---|---|
| | n | (%) | n | (%) |
| ***Entropion*** | | | | |
| None | 98 | (74·8) | 60 | (65.9) |
| Mild | 28 | (21·4) | 21 | (23.1) |
| Moderate | 4 | (3·0) | 10 | (11.0) |
| Severe | 1 | (0·8) | 0 | (0.0) |
| ***Trichiatic eyelash burden*** | | | | |
| 0 (epilating) | 20 | (15·3) [a] | 7 | (7.7) [b] |
| 1 | 42 | (32·1) | 46 | (50.5) |
| 2 | 27 | (20·6) | 20 | (22.0) |
| 3 | 19 | (14·5) | 6 | (6.6) |
| 4 | 10 | (7.6) | 4 | (4.4) |
| 5 | 5 | (3.8) | 6 | (6.6) |
| 6 + | 8 | (6.1) | 2 | (2.2) |
| ***Trichiatic eyelash type*** | | | | |
| Epilating | 20 | (15·3) | 7 | (7.7) |
| Entropic | 10 | (7·6) | 8 | (8.8) |
| Metaplastic | 82 | (62·6) | 67 | (73.6) |
| Misdirected | 8 | (6·1) | 7 | (7.7) |
| Mixed | 11 | (8.4) | 2 | (2.2) |

*[a] Among the 20 PTT cases who had evidence of successful epilation, 14 (70%) epilated <1/3rd of the eyelid margin length, 5 (25%) epilated 1/3rd – 2/3rd of the eyelid margin length and 1 (5%) epilated >2/3rd of the eyelid margin length.*
*[b] Among the 7 PTT cases who had evidence of successful epilation 6 (85.7%) epilated <1/3rd of the eyelid margin length and 1 (14.3%) epilated 1/3rd – 2/3rd of the eyelid margin length.*

*N = trial participants who were identified with PTT at the 12-month follow-up.*


1. Habtamu E, Wondie T, Aweke S, Tadesse Z, Zerihun M, Zewudie Z, et al. Posterior lamellar versus bilamellar tarsal rotation surgery for trachomatous trichiasis in Ethiopia: a randomised controlled trial. The Lancet Global Health. 2016;4(3):e175-e84. doi: 10.1016/S2214-109X(15)00299-5.
2. Habtamu E, Wondie T, Aweke S, Tadesse Z, Zerihun M, Gashaw B, et al. Oral doxycycline for the prevention of postoperative trachomatous trichiasis in Ethiopia: a randomised, double-blind, placebo-controlled trial. The Lancet Global Health. 2018;6(5):e579-e92. doi: 10.1016/S2214-109X(18)30111-6.
