# Supplementary figures and images for: Effect of repeated epilation for minor trachomatous trichiasis on lash burden, phenotype and surgical management willingness: A cohort study
Source: PLoS Negl Trop Dis. 2020 Dec 14;14(12):e0008882. doi: 10.1371/journal.pntd.0008882 (PMC7769600; doi:10.1371/journal.pntd.0008882)

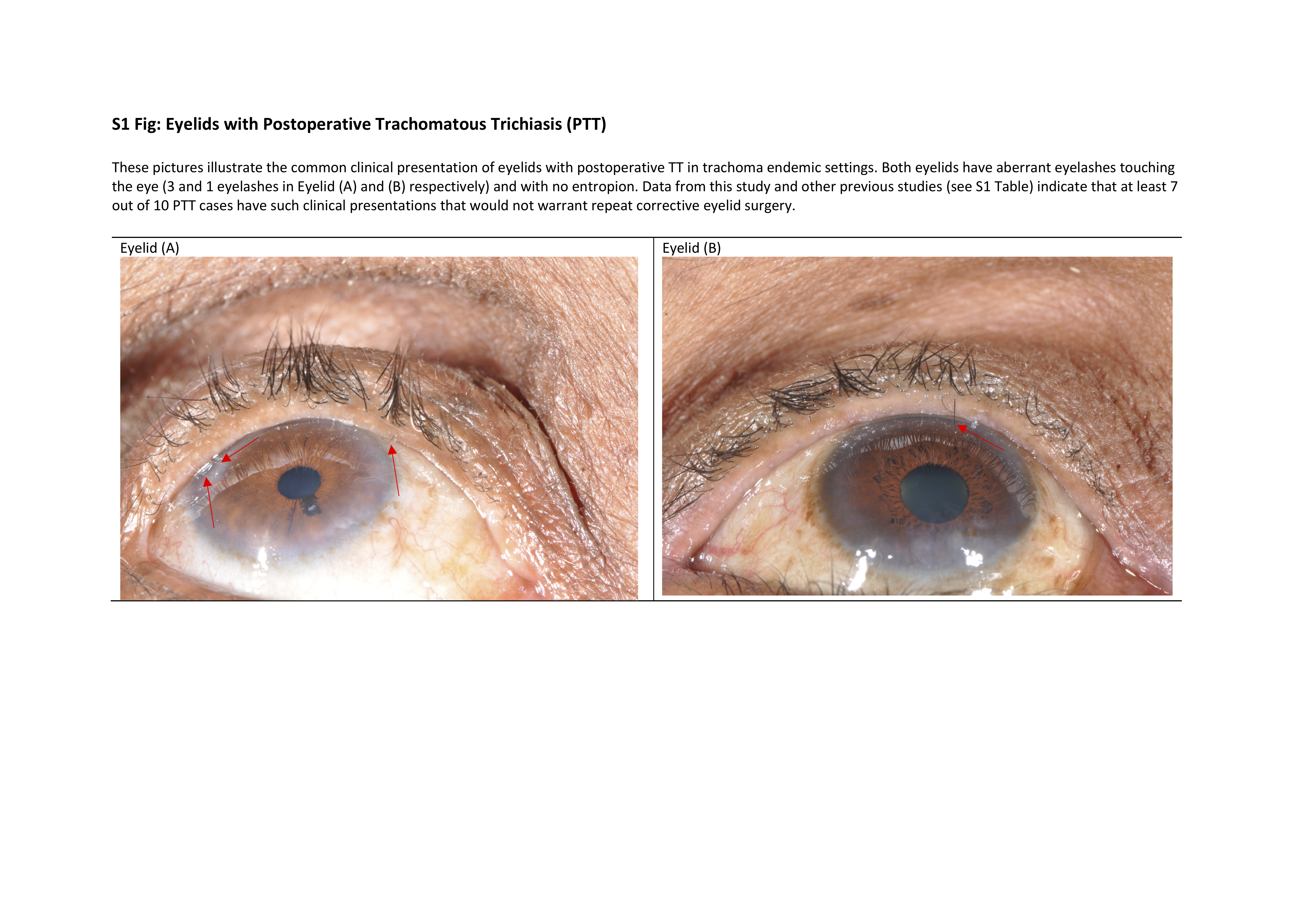

Supplement: S1 Fig — (TIFF) [file pntd.0008882.s002.tiff]
